# Short-run marginal emission rates omit important impacts of electric-sector interventions

**DOI:** 10.1073/pnas.2211624119

**Published:** 2022-11-28

**Authors:** Pieter J. Gagnon, John E. T. Bistline, Marcus H. Alexander, Wesley J. Cole

**Affiliations:** ^a^Grid Planning and Analysis Center, National Renewable Energy Laboratory, Golden, CO 80401; ^b^Energy Systems and Climate Analysis Group, Electric Power Research Institute, Palo Alto, CA 94304; ^c^Electric Transportation Group, Electric Power Research Institute, Palo Alto, CA 94304

Holland et al. (1) describe the calculation of a marginal emission rate and advocate for using such metrics when estimating the impacts of electric-sector policies and interventions. Importantly, however, what is being calculated in their study (1) is a short-run marginal emission rate, meaning that it characterizes the marginal emissions from a fixed electricity system, neglecting the impacts of any structural change that may be induced by the intervention.

Why does this matter? It is because the current policy and economic landscape in the United States means that new electric load would often induce more nonemitting generators, such as wind and solar, to be built (2, 3). The short-run mixture (which is dominated by natural gas and coal) can look quite different from the long-run mixture that includes newly built generators. This is especially relevant for the 31 states (and DC) with electricity portfolio standards, which require a portion of generation to be clean, often necessitating structural responses to load increases.

The omission of induced structural change from short-run methodologies was recognized previously by a subset of the authors of ref. 1. When discussing the limitations of the short-run method employed in ref. 4, for example, the appendix recognizes that “A full model would need to account for entry and exit of power plants and transmission capacity.” A similar caveat was given in ref. 5.

Such caveats were warranted because neglecting induced structural change can be impactful (6, 7). Holland et al. (1) calculated a marginal CO_2_ emission rate of 591 kg/MWh for the United States in 2019 and applied that to the Biden administration’s vehicular electrification targets through 2030. By contrast, if we take into account induced structural change with long-run marginal emission rates (8), we estimate the policy’s impact as 286–336 kg/MWh across three “business-as-usual” futures—reflecting the notion that if we electrify vehicles, we are likely to build more generators, many of which will be nonemitting.

**Fig. 1.**
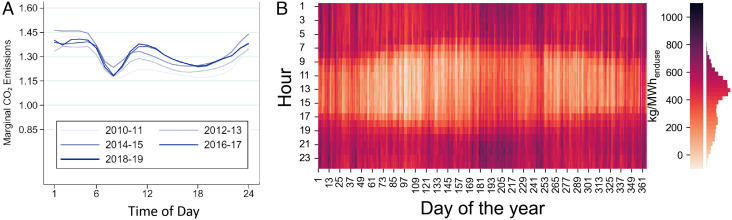
Examples of time-varying trends in short-run and long-run marginal emission rate approaches. Panel *A* shows short-run marginal emission rates from ref. 1 in units of lb/kWh of busbar load for 5 historical years. Panel *B* shows long-run marginal emission rates from ref. 6 in kg/MWh of end-use load for 2024–2043. Both panels give data for the contiguous United States.

Additionally, long-run analysis can show diurnal and seasonal trends that are not captured through short-run approaches (Fig. 1). Motivated by this, if we assume that the new electric vehicles are mostly charged during the day, we see an even lower estimate of 200–286 kg/MWh. More flexible or targeted charging strategies could achieve even lower emission rates—Gagnon and Cole (6) saw an afternoon load addition with a negative emission rate, for example. Long-run marginal rates have their own limitations as well (partially discussed in ref. 6 and the documentation for (8), although much work remains to be done), of course, but help us here to gain a sense of the potential magnitude of structural impacts.

Recognizing its importance, leading organizations employ methods that incorporate structural impacts (9, 10).

To be clear, we agree with Holland et al. (1) regarding the value of marginal approaches when estimating the impacts of interventions—but we append that the methods would ideally capture the interventions’ effects comprehensively. Short-run metrics do not do so, and by neglecting induced structural change, they can erroneously estimate the consequences of actions. This undermines the promise of marginal emission rates to support decision-making, distorting the selection between alternatives based on their impacts (Table 1).

**Table 1.** Differences in estimates of electric-sector emissions induced by the Biden administration’s electric vehicle targets when short-run and long-run approaches are used

Source	Factor type	Charging strategy	Marginal emission factor (kg/MWh)
Holland et al. 2022	Short-run	Unmanaged*	591–631
Cambium 2021	Long-run	Unmanaged*	286–336
Cambium 2021	Long-run	Mostly daytime**	200–286

^*^Unmanaged charging strategies assume that EV load is added proportionally to existing loads.

^**^The mostly daytime charging strategy assumes that 90% of EV load is added from 8 am to 4 pm.
